# Antibiotic use in pediatric acute care hospitals: an analysis of antibiotic consumption data from Germany, 2013–2020

**DOI:** 10.1007/s15010-023-02112-w

**Published:** 2023-11-02

**Authors:** Mirjam Freudenhammer, Markus Hufnagel, Michaela Steib-Bauert, Ulrich Mansmann, Katja de With, Matthias Fellhauer, Winfried V. Kern

**Affiliations:** 1 Division of Pediatric Infectious Diseases and Rheumatology, Department of Pediatrics and Adolescent Medicine, University Medical Center Freiburg, Freiburg, Germany; 2 Center for Chronic Immunodeficiency, Institute for Immunodeficiency, University Medical Center Freiburg, Freiburg, Germany; 3 Division of Infectious Diseases, Department of Internal Medicine II, University Medical Center Freiburg, Freiburg, Germany; 4 Faculty of Medicine, University of Freiburg, Freiburg, Germany; 5 Institute for Medical Information Processing, Biometry and Epidemiology, Faculty of Medicine, LMU Munich, Munich, Germany; 6 Division of Infectious Diseases, University Hospital Carl Gustav Carus Dresden at the TU Dresden, Dresden, Germany; 7 Pharmacy/Institute for Clinical Pharmacy, Schwarzwald-Baar Hospital, Villingen-Schwenningen, Germany

**Keywords:** Antibiotic consumption, Pediatric hospitals, Pharmacy prescription data, Defined daily dose, Recommended daily dose

## Abstract

**Background:**

Antimicrobial stewardship (AMS) programs are effective tools for improving antibiotic prescription quality. Their implementation requires the regular surveillance of antibiotic consumption at the patient and institutional level. Our study captured and analyzed antibiotic consumption density (ACD) for hospitalized pediatric patients.

**Method:**

We collected antibacterial drug consumption data for 2020 from hospital pharmacies at 113 pediatric departments of acute care hospitals in Germany. ACD was calculated as defined daily dose (DDD, WHO/ATC Index 2019) per 100 patient days (pd). In addition, we analyzed the trends in antibiotic use during 2013–2020.

**Results:**

In 2020, median ACD across all participating hospitals was 26.7 DDD/100 pd, (range: 10.1–79.2 DDD/100 pd). It was higher at university vs. non-university hospitals (38.6 vs. 25.2 DDD/100 pd, *p* < 0.0001). The highest use densities were seen on oncology wards and intensive care units at university hospitals (67.3 vs. 38.4 DDD/100 pd). During 2013–2020, overall ACD declined (− 10%) and cephalosporin prescriptions also decreased (− 36%). In 2020, cephalosporins nevertheless remained the most commonly dispensed class of antibiotics. Interhospital variability in cephalosporin/penicillin ratio was substantial. Antibiotics belonging to WHO AWaRe “Watch” and “Reserve” categories, including broad-spectrum penicillins (+ 31%), linezolid (+ 121%), and glycopeptides (+ 43%), increased over time.

**Conclusion:**

Significant heterogeneity in ACD and prescription of different antibiotic classes as well as high prescription rates for cephalosporins and an increased use of reserve antibiotics indicate improvable antibiotic prescribing quality. AMS programs should urgently prioritize these issues to reduce antimicrobial resistance.

**Supplementary Information:**

The online version contains supplementary material available at 10.1007/s15010-023-02112-w.

## Introduction

Increasingly, antimicrobial resistance has emerged as a global health problem—one linked to worse patient outcomes and increasing healthcare costs [1, 2]. Several studies have drawn connections between inappropriate antibiotic use and the development of antimicrobial resistance caused by selection pressure on both bacterial pathogens and on human flora microorganisms [1, 3, 4]. Antimicrobial resistance can develop after even a single dose of antibiotics and can persist for months afterward [5]. Furthermore, prolonged antimicrobial use is associated with toxicity. Even so, in up to 50% of cases, antimicrobials are prescribed inappropriately, either for treatment of infections or else as prophylaxis [6, 7]. For these reasons, it is urgent that antimicrobial use become managed more prudently. In 2015, a call for optimization of their use became part of the World Health Organization's (WHO) “Global action plan on antimicrobial resistance” [8]. Commonly labeled antimicrobial stewardship (AMS) programs, initiatives to improve antimicrobial prescribing have been developed. These have been proven effective also in pediatric settings in reducing antimicrobial prescription rates and related costs, as well lowering usage of reserve antibiotics and the antimicrobial resistance that accompanies it [9–14].

Besides reducing the overall antibiotic prescription rates by avoiding not-indicated or prolonged antibiotic treatments, the WHO emphasized on using antibiotics with a low risk for antimicrobial resistance whenever possible and classified antibiotics accordingly into the categories “Access”, “Watch”, and “Reserve” [15]. To reduce development of antimicrobial resistance, 60% of the total antibiotic consumption should consist of antibiotics belonging to access group antibiotics [16].

Crucial to every AMS program is the regular collection of antibiotic consumption data and the audit of antibiotic prescribing to identify potentially inappropriate prescribing and subsequently ameliorate it. Although the overall quantity of antibiotics prescribed to hospitalized children in Germany is small, the density of antibiotic consumption in this setting is high; 30–40% of children hospitalized in Germany receive at least one antibiotic for either therapy or prophylaxis [7, 17]. Unfortunately, a pool of regularly-collected, antibiotic consumption data is not available from pediatric hospitals in Germany. Therefore, the aim of our study was to analyze available antibiotic consumption data from the ADKA-if-DGI project (http://www.antiinfektiva-surveillance.de) in relation to hospital size and service type and to then record changes observed over the surveillance period 2013–2020.

## Methods

Data were collected from German pediatric hospitals with acute care wards who were participants in the ADKA-if-DGI project. Quarterly hospital pharmacy reports on the quantity (number of units) of systemic antibiotics (oral or parenteral) dispensed to various wards or departments with unique cost centers were logged and converted into "Defined Daily Doses" (DDD, WHO/ATC Index 2019) [18]. Bed occupancy data were collected from the local hospital administration. Antibiotic consumption density (ACD) was expressed as DDD per 100 patient days, (pd, i.e., occupied bed days). Results were additionally calculated by employing “Recommended Daily Dose” (RDD) definitions of hospital-adapted doses for a variety of drugs that better reflect truly prescribed doses in hospitalized (adult) patients [19]. Details on data collection and analysis in the ADKA-if-DGI project have been described previously [20].

For the cross-sectional analysis, we calculated median and interquartile ranges (25% and 75% percentiles). Pooled means per year were used to describe longitudinal changes. Data were derived from general wards, intensive care wards (pediatric and/or neonatal ICUs), and hematology–oncology wards. Hospitals were divided into groups according to overall size (including non-pediatric beds): small hospitals (< 400 beds), medium-sized hospitals (400–800 beds), and large hospitals (> 800 beds). Among the large hospitals, university hospitals were evaluated separately. Data were compared using either the Wilcoxon rank-sum test for comparison of two groups, or else the Kruskal–Wallis test for comparison of more than two groups. All statistical tests were calculated with GraphPad Prism V.6 (GraphPad Software, La Jolla, CA, USA). These tests were two-tailed and considered significant if the p value was < 0.05. We also performed a panel analysis (several cross-sectional over several timepoints) using linear mixed effect models and by random for institutional effects in effects intercept and change over time [21]. Each hospital was coded by a random effect and time. Other co-variates such as hospital size and category as well as ward type were used to estimate antibiotic use over time and how this may be influenced by relevant factors. Comparisons between different antibiotic classes were done descriptively using generalized forest plots. This analysis used the Software R (Version 4.0.3. 2020-10-10 [22]) RStudio [23] and the packages lme4 [24] and metafor [25]. For assignment of antibiotic substances to displayed antibiotic classes and groups, see Supplementary Table 1. To describe consumption of cephalosporins, the consumption of 1st/2nd generation cephalosporins and 3rd/4th generation cephalosporins was added up. The consumption of penicillins was computed as sum of broad-spectrum penicillins, aminopenicillins/beta-lactamase inhibitors (BLI), and narrow-spectrum penicillins. For classification of antibiotics to WHO AWaRe groups, the WHO 2021 AWaRe classification was employed [15].

## Results

### Antibiotic consumption density in 2020

During 2020, a combination of 113 children's hospitals and pediatric divisions of acute care hospitals in Germany submitted a full year (four quarters) of antibiotic consumption data covering a total of 1,693,501 pd. Among the reporting hospitals were 36 hospitals with < 400 beds (32%), 36 with 400–800 beds (32%), and 41 (including 22 university hospitals) with > 800 beds (36%) (Table 1).

**Table 1:** Comparison of antibiotic consumption density in DDD and RDD per 100 patient days between university and non-university hospitals of different sizes for the year 2020

	*n*	DDD/100 pd		RDD/100 pd	
		Median	Interquartile range	Median	Interquartile range
Non-university hospitals	91	25.25	19.05–31.27	18.65	13.58–22.90
< 400 beds	36	22.69	18.37–32.25	17.80	12.18–23.77
400–800 beds	36	23.68	18.56–27.28	18.48	13.59–20.79
> 800 beds	19	28.52	24.05–34.95	22.29	19.38–26.48
University hospitals (> 800 beds)	22	38.64	29.42–43.77	31.13	25.10–36.79

pd, patient days

**Fig. 1:**
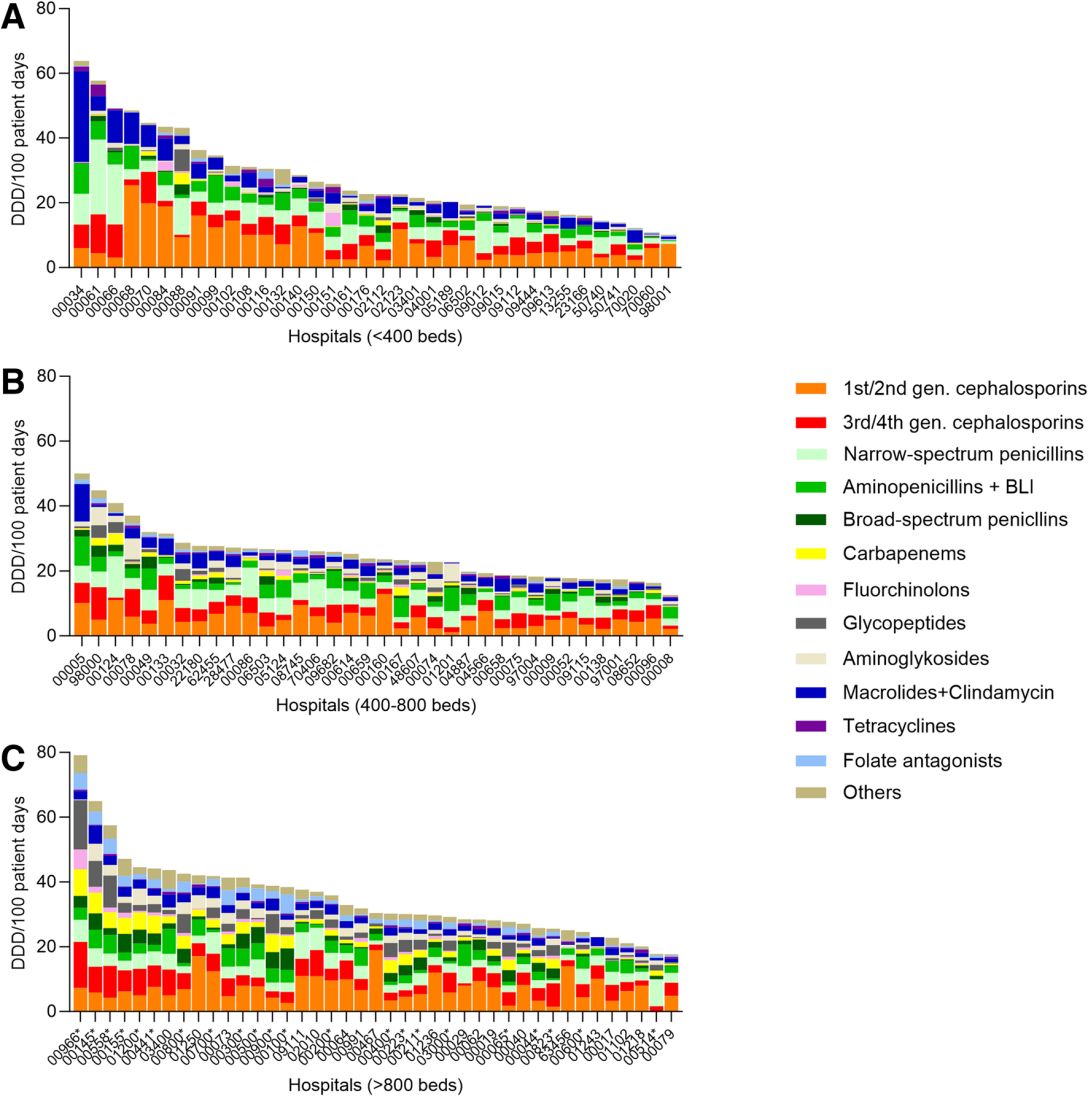
Antibiotic consumption density (in DDD/100 patient days) of specific antibiotic classes at 113 pediatric hospitals of different sizes and categories. **A** Hospitals with < 400 beds, **B** hospitals with 400–800 beds, **C** hospitals with > 800 beds. University hospitals are marked with a asterisk. Data are for the year 2020. BLI, beta-lactamase inhibitors

Median overall ACD at pediatric divisions of acute care hospitals was 26.7 DDD/100 pd, with an interquartile range of 20.1–36.3 (or median 20.7 RDD/100 pd, IQR 15–26.4) (Supplementary Table 2). Significantly higher ACD was found at university vs. non-university hospitals (*p* < 0.0001). At non-university hospitals, ACD increased in parallel with hospital size (Table 1). However, variability between individual hospitals, (which ranged from 10.1 to 79.2 DDD/100 pd), was high—a span corresponding to 7.8-fold difference among hospitals (Fig. 1). Interestingly, substantial variability in overall antibiotic use was also seen among hospitals of similar size (Fig. 1). For example, the difference in overall ACD among the 22 university hospitals was approximately 4.4-fold (79.2 DDD/100 pd, resp. 17.8 DDD/100 pd).

ACD was especially high on intensive care units (ICUs) and hematology/oncology wards of university hospitals (Table 2 and Supplementary Table 3). Here, the greatest disparity between university hospitals and non-university hospitals was observed. By contrast, on regular wards, ACD was similar for both university and non-university hospitals. Again however, increased ACD was observed to increase along with size of the non-university hospital on ICUs and regular wards.

**Table 2:** Comparison of antibiotic consumption density in DDD/100 patient days between different wards (regular, ICU, and hematology–oncology) in hospitals of different size (< 400, 400–800, > 800 beds) and of different categories (non-university and university hospitals) in the year 2020

	Non-university hospitals			University hospitals			*p* value*
	*n*	Median	Interquartile range	*n*	Median	Interquartile range	
Regular wards	90	28.51	22.27–34.17	21	29.17	23.48–39.14	0.2988
> 800 beds	19	31.70	27.82–36.79	21	29.17	23.48–39.14	0.6878
400–800 beds	35	27.09	21.92–33.17				
< 400 beds	36	26.79	20.09–31.53				
ICU	53	15.40	8.56–25.22	22	38.94	31.37–49.98	0.0001
> 800 beds	15	19.47	10.66–31.76	22	38.94	31.37–49.98	0.0003
400–800 beds	27	15.98	10.07–24.07				
< 400 beds	11	8.80	6.04–11.98				
Hematology/oncology	3	31.98	26.19–42.91	18	67.28	59.32–86.40	0.0105

*Wilcoxon rank-sum test

### Antibiotic classes

The five most common classes of antibiotics across all hospitals and wards in 2020 were (in DDD/100 pd): 1st/2nd generation cephalosporins, narrow-spectrum penicillins, 3rd/4th generation cephalosporins, aminopenicillins/BLI combinations, and macrolides/clindamycin (for RDD, see Supplementary Table 2). As with overall ACD, interhospital variability regarding prescription of different antibiotic classes was substantial (Fig. 1). This variability encompassed differences between cephalosporin and penicillin consumption (ratio ranging between 15:85 and 87:13), as well as that between piperacillin ± BLI and ampicillin/amoxicillin ± BLI, and that between 1st/2nd generation and 3rd/4th generation cephalosporins (Fig. 1).

**Fig. 2:**
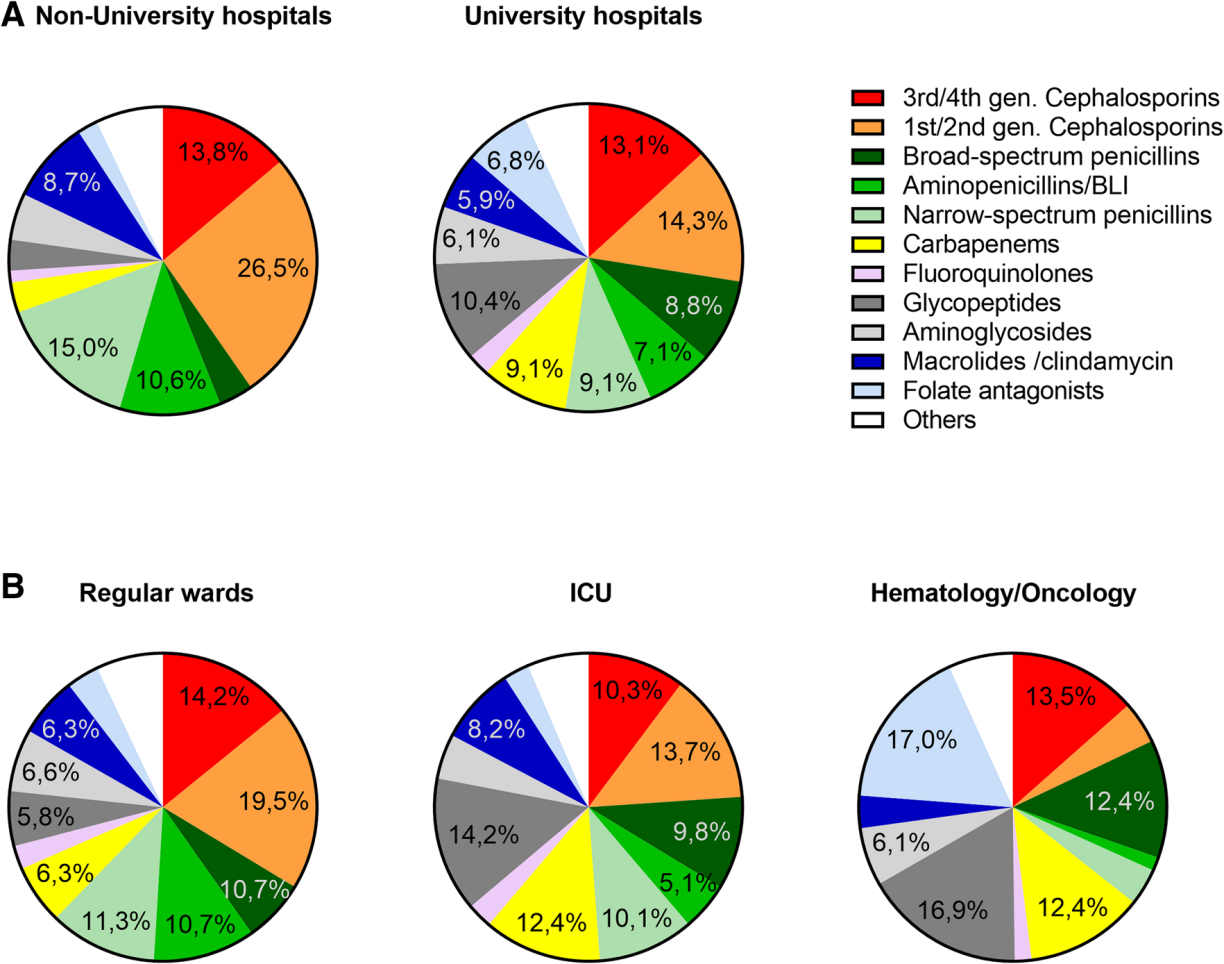
Use of antibiotic classes in patients in non-university and university hospitals (all wards) (**A**) and on regular hospital wards vs. ICU and oncologic wards of university hospitals (**B**) as a proportion of the median antibiotic consumption density (in % DDD) in the respective stratum. Data for the year 2020. BLI, beta-lactamase inhibitors

As part of overall antibiotic consumption (in DDD), the proportion of broad-spectrum beta-lactams, (carbapenems, broad-spectrum penicillins and 3rd/4th generation cephalosporins), as well as their median ACD in DDD/100 pd, was significantly higher at university hospitals (31%; 11.2 DDD/100 pd) vs. non-university hospitals (21%; 4.6 DDD/100 pd (Fig. 2A and Supplementary Table 4). In comparing cephalosporin and penicillin consumption, however, the ratio remained similar between university (53:47) and non-university hospitals (57:43)—49:51 and 53:47 when calculated with RDD, respectively.

As expected, consumption of reserve and broad-spectrum antibiotics such as carbapenems, glycopeptides, and broad-spectrum penicillins was higher in ICU (especially at university hospitals) and oncology wards vs. in patients on regular wards. This is reflected in median DDD/100 and RDD/100, as well as in proportion of overall antibiotic use (Fig. 2B, Supplementary Table 4, and data not shown). For example, median usage of glycopeptides on ICUs and oncology wards at university hospitals was 30-fold and 49-fold higher than on regular wards at non-university hospitals (DDD/100 pd: 6.25 vs. 10.39 vs. 0.21, respectively).

### AWaRe classification

**Fig. 3:**
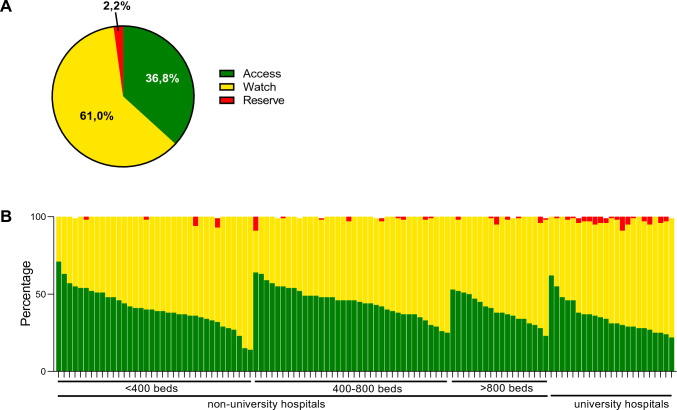
Percentage of antibiotic use in children according to the WHO AWaRe classification for pediatric hospitals with acute care wards (**A**) and as individual hospitals (**B**). Data for the year 2020

According to the WHO AWaRe classification 2021, 63% of the antibiotics used during 2020 (in DDD) belong to the “Watch” and “Reserve” categories (Fig. 3A). While use of antibiotics from these categories varied extensively among individual hospitals (29 – 86%, Fig. 3B), higher consumption was observed in university vs. non-university hospitals (median 69.0% vs. 58.5%, *p* = 0.0013).

### Longitudinal surveillance 2013–2020

The number of hospitals submitting data increased from 66 in 2013 to 113 in 2020, with 48 of the hospitals submitting data during every year of the surveillance period. From 2013 to 2020, overall ACD in DDD/100 pd decreased by 10% (*p* < 0.0001), a significant change (Table 3) observed at university and non-university hospitals (Fig. 5), as well as at hospitals of varying sizes (data not shown). The most notable decline was observed on hematology/oncology wards (*p* = 0.03)—the setting with the highest overall antibiotic consumption.

**Table 3:** Evolution of overall antibiotic consumption density and use of single antibiotic classes during the course of the surveillance period 2013–2020. Statistics were calculated using a linear mixed model

	Pooled mean (DDD/100 pd)								Change (%) 2013 to 2020	Regression coefficient	Confidence intervals		*p* value
	2013	2014	2015	2016	2017	2018	2019	2020					
	(*n* = 66)	(*n* = 70)	(*n* = 72)	(*n* = 79)	(*n* = 93)	(*n* = 104)	(*n* = 110)	(*n* = 113)			2.5%	97.5%	
Overall consumption	35.91	33.52	35.68	35.13	32.28	32.21	33.15	32.32	-9.99	-0.7133	-1.00	-0.43	< 0.0001
3^rd^/4^th^ gen. cephalosporins	4.67	4.30	4.41	4.36	3.97	4.06	4.14	4.35	-6.73	0.0105	-0.05	0.07	0.7147
Broad-spectrum penicillins	5.97	6.23	6.66	6.78	6.35	6.70	7.00	7.83	+ 31.09	0.0826	0.07	0.10	< 0.0001
Carbapenems	1.60	1.61	1.71	1.80	1.79	1.81	1.87	2.10	+ 31.50	0.0216	0.01	0.04	0.0106
1^st^/2^nd^ gen. cephalosporins	12.01	10.28	10.61	10.19	8.51	7.75	7.50	6.37	-47.00	-0.8957	-1.03	-0.76	< 0.0001
Aminopenicillins/BLI	1.62	1.60	1.79	1.93	1.99	2.37	2.85	2.80	+ 72.77	0.2238	0.19	0.26	< 0.0001
Narrow-spectrum penicillins	3.36	3.03	3.46	3.98	3.52	3.80	4.20	3.78	+ 12.43	0.0931	0.01	0.17	0.0229
Fluoroquinolones	0.95	1.06	1.08	0.95	0.96	0.89	0.75	0.60	-37.16	-0.0405	-0.06	-0.03	< 0.0001
Glycopeptides	1.64	1.80	1.84	1.89	1.96	2.05	2.17	2.34	+ 43.36	0.0172	0.00	0.04	0.0830
Aminoglycosides	1.79	1.73	1.94	1.87	1.75	1.68	1.68	1.79	+ 0.07	-0.0309	-0.07	0.01	0.1538
Macrolides /clindamycin	3.77	3.05	3.40	3.06	2.62	2.52	2.57	2.31	-38.71	-0.1662	-0.23	-0.10	< 0.0001
Tetracyclines	0.44	0.53	0.51	0.45	0.49	0.42	0.42	0.37	-15.61	-0.0154	-0.03	0.00	0.0579
Folate antagonists	1.62	1.67	1.68	1.46	1.60	1.52	1.50	1.54	-4.87	-0.0303	-0.05	-0.01	0.0012
Linezolid	0.13	0.12	0.12	0.15	0.17	0.19	0.21	0.28	+ 121.4	0.0096	-0.05	-0.01	0.0003
Metronidazole	1.22	1.23	1.35	1.26	1.20	1.17	1.18	1.22	+ 0.63	-0.0046	-0.03	0.02	0.6913
Penicillins	5.76	5.86	6.69	7.40	6.90	7.78	8.78	8.67	+ 50.54	0.4004	0.30	0.50	< 0.0001
Cephalosporins	16.68	14.57	15.02	14.55	12.48	11.81	11.64	10.72	-35.73	-0.8864	-1.04	-0.73	< 0.0001

pd, patient days

**Fig. 4:**
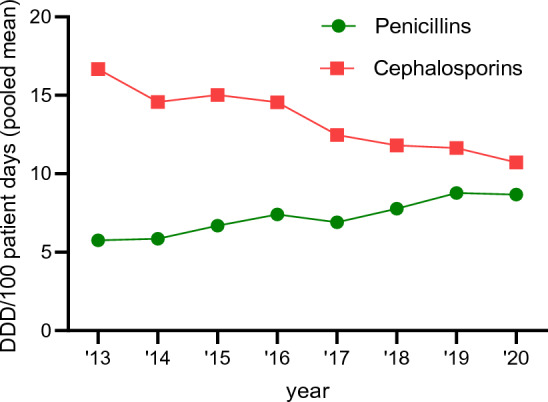
Antibiotic consumption density of penicillins and cephalosporins (DDD/100pd) for the time period 2013–2020

**Fig. 5:**
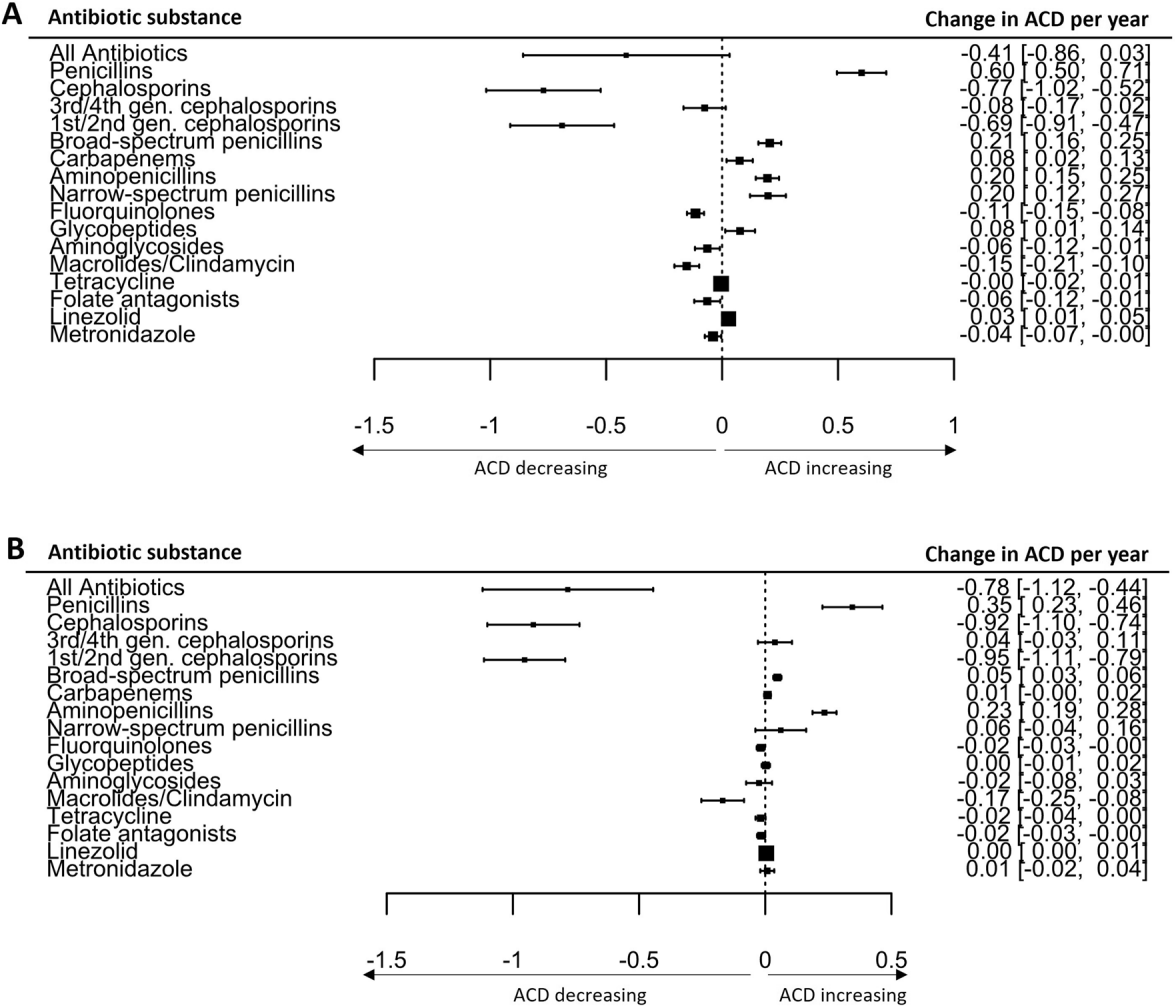
Overall antibiotic consumption density and consumption of single antibiotic classes as usage evolved during the course of the surveillance period (2013–2020) at university hospitals (**A**) and non-university hospitals (**B**). Data shown as a regression coefficient representing the change in ACD per year with 2.5%/97.5% confidence intervals. Statistics were calculated using a linear mixed model adjusted for hospital service type

Consumption of penicillins increased significantly (+ 50.5%, *p* < 0.0001), whereas cephalosporins declined (− 35.7%, *p* < 0.0001, Fig. 4). Use of macrolides/clindamycin, tetracycline, and fluoroquinolones decreased over the 8-year period, while prescription of “reserve antibiotics” increased, especially at university hospitals (Fig. 5). These reserve antibiotics included broad-spectrum penicillins (piperacillin–tazobactam), carbapenems, glycopeptides, and linezolid (Table 3).

## Discussion

Our study collected and analyzed nationwide ACD at pediatric hospitals with acute care wards in Germany.

Overall ACD was 26.7 DDD/100 pd, with 25.3 DDD/100 pd for non-university and 38.6 DDD/100 pd for university hospitals in 2020. In comparison to findings from other European countries, this is on the lower range [26–29]. Variability of antibiotic use among the participating hospitals was striking, with a 7.8-fold difference in antibiotic use among the hospitals that had the lowest and the highest ACDs (Fig. 1). The higher ACD found in larger hospitals and in university hospitals vs. that seen in smaller hospitals and non-university hospitals may be explained by several factors: larger hospitals with > 800 beds mostly belong to tertiary care centers with specialized pediatric care units, and therefore treat many patients with underlying diseases (e.g., immunodeficiency, cystic fibrosis, post-kidney transplant, etc.). These patients are more prone to develop severe infections, to develop hospital-acquired infections, to be colonized by resistant microorganisms and to receive (broad-spectrum) antibiotics, factors that influence the ACD. Moreover, patients with severe infections/underlying conditions are often transferred to larger and university hospitals. In addition, hematology/oncology or bone marrow transplant wards, that were shown to have an extensively high antibiotic consumption, as well as NICUs caring for extremely low birth weight infants, are exclusively located in large (university) hospitals. Other institutional differences between small and large/non-university and non-university hospitals that may influence antibiotic prescription and consumption include existence of on-site infectious disease (ID) consulting services and/or AMS teams. Nevertheless, we also observed meaningful differences in ACD among participating university hospitals or between small hospitals with less than 400 beds, assuming comparable patient populations and treatment indications among these hospitals. This within-group variability suggests there to be either suboptimal adherence to national guidelines or else the existence of suboptimal local guidelines, ones that likely would benefit from the institution of a local AMS program [9, 14].

Several point or period prevalence surveys (PPS), that describe antibiotic prescribing practice at patient level, confirm our finding of a high variability in antibiotic consumption. In 2012, as part of the Antibiotic Resistance and Prescribing Project (ARPEC), a PPS from 23 German pediatric hospitals reported a similarly high variation in antibiotic prescribing practices [17, 30]. Here, antibiotic prevalence rates (APR) ranged from 6.5 to 49.5% (a 7.6-fold difference) among surveyed hospitals. The highest APR were recorded on oncology wards (65.0%), while the lowest were on general neonatal wards (7.3%). APR was higher at university hospitals (27.0%) as compared to non-university hospitals (16.7%). A British PPS study that examined the proportion of children who had been prescribed antibiotics, along with the DDD/100 pd per age group, also reported a wide variation in antibiotic use when comparing practices at the district general hospital and tertiary referral hospitals, as well as when examining the two types of hospitals separately [29].

Our study's finding that antibiotic use on hematology/oncology wards and ICUs was higher than on regular pediatric wards is in line with the results reported from several multicenter PPS from Europe and elsewhere [17, 29–33]. This finding may be explained by the high percentage of fever and severe infections, as well as the need for antimicrobial prophylaxis, in critically ill and/or vulnerable patient populations. Moreover, children who are on hematology/oncology/transplant wards or on ICUs are more likely to receive antibiotic combination therapies, a factor directly affecting the DDD/100 pd [17, 29]. However, several studies have shown that approximately half of antibiotic treatments on pediatric ICUs (PICUs) were inappropriate [32, 34, 35] and that significant reductions in antimicrobial use can be achieved on PICU and hematology/oncology wards (without increased adverse outcomes), when effective AMS programs, e.g., prospective audit with feedback or preauthorization systems, are implemented [36–40].

Assuming a similar case mix index and age structure in the participating hospitals over the surveillance period 2013–2020, our finding that antibiotic use among participating hospitals decreased over time is both noteworthy and relevant. Of particular interest is that the use of cephalosporins decreased by approximately 36%, whereas the usage of penicillins increased by 51%. According to the WHO AWaRe classification of antibiotics, penicillins are the preferred class of antibiotics for most pediatric indications and should replace 2nd, 3rd, or 4th generation cephalosporins whenever possible [41]. The use of cephalosporins is associated with the development of *C. difficile* infections (CDI) [42], as well as with the emergence of antibiotic-resistant microorganisms, including extended-spectrum beta-lactamase-producing Gram-negative bacteria [43, 44] and vancomycin-resistant enterococci [45]. The observed change in consumption density of penicillins and cephalosporins during the period 2013–2020 may be related to AMS initiatives successfully implemented in many German hospitals or the increasing availability of pediatric-specific antibiotic stewardship education programs—a positive development [10, 39, 46–48]. Unfortunately, however, our study found that in 2020, in both university and non-university hospitals, cephalosporins remained the most commonly used antibiotics across all pediatric departments (Table 3 and Supplementary Tables 2 and 4). This stayed true when RDD/100 pd was used as an alternative metric instead of DDD/100 pd, as DDD is based on a lower daily dose for cephalosporins, and therefore overestimates their use. In line with our conclusions, high consumption of cephalosporins (especially 3rd generation cephalosporins) has been identified via PPS in several other European countries as well. This emphasizes the need for a comprehensive, international effort to address antibiotic prescription quality [17, 49–53]. In some pediatric hospitals in Europe, including in Sweden, cephalosporin use has been successfully reduced over a 15-year period [54].

Despite overall decreases in antibiotic use (including that of cephalosporins), increased consumption of antibiotics belonging to the WHO “Watch” or “Reserve” categories—specifically, carbapenems, broad-spectrum penicillins, glycopeptides and linezolid — is concerning and needs further monitoring. According to a recent global PPS from the Global Antimicrobial Resistance, Prescribing, and Efficacy in Neonates and Children (GARPEC) and the Global PPS on Antimicrobial Consumption and Resistance (Global-PPS) networks, antibiotics belonging to the WHO “Watch” category in Europe and in Germany (five participating hospitals) were prescribed to pediatric patients at a rate of approximately 40% [51]. In our study, 61% of the dispensed antibiotics (in DDD) were antibiotics from the “Watch” category, despite substantial variations among hospitals. This misses the WHO target—a goal saying that at least 60% of total antibiotic consumption be from “Access” group antibiotics—by a long shot [16]. AMS activities as facility-specific treatment recommendations (including indication, choice of substance and duration of antibiotic treatment) or preauthorization systems for antibiotics belonging to “Watch” and “Reserve” categories might help to limit the extensive use of antibiotics from these categories [55, 56]. With 113 out of a total of 318 German pediatric hospitals participating during 2020 [57], our sample, which included both small and large hospitals, is of considerable size (36%). Because we do not have information on the specific populations served by each hospital participating in the study, we unfortunately are unable to determine the extent to which the selection of hospitals may be fully representative of all pediatric hospitals in Germany. Assessing antibiotic consumption as drug dispensing data in the form of DDD has its limitations, especially in pediatric populations as DDD usually represent average doses for adult patients in the community or hospital, rather than those effectively prescribed at the patient level, e.g., as days of therapy (DOTs) [58–60]. Therefore, no statements can be made regarding the quality of the single antibiotic prescription.

Moreover, although RDD/100 pd is closer to the actual dose of some antibiotics than DDD/100 pd in adult patients [61], calculations of both DDD and RDD are based on the average maintenance dose per day in adults and do not consider individual pediatric prescribing practices based upon age and/or body weight or body surface area (BSA). When dispensed by the pharmacy, either only a fraction of the antibiotic dose is given to the patient and the rest discarded, or else the dose might be divided among several pediatric patients. Correlation of DDD/RDD with actual consumption is dependent upon body weight variations in the studied population, upon the different drug vial sizes available at the hospital pharmacy, and upon the percentage of discarded drugs [62–64]. Therefore, DDD and RDD do not accurately reflect actual drug consumption on pediatric wards. Both overestimation and underestimation of drug use are of concern [33, 65, 66]. Furthermore, due to heterogeneity in patient age and body weight, as well as to variations in dosing schemes, comparability between wards and institutions might be impacted [59]. Metrics considered more appropriate for the capture of antibiotic consumption data in children include days of therapy, length of therapy or prescribed daily dose. Unfortunately, these data were not available to our study, as they generally require access to the individual patient, and collecting such data on a regular basis requires use of electronic health records to be cost-effective, which to date are not nationwide available [33, 47, 59, 66–68]. Nonetheless, given the variability of ACD even among university hospitals, which is less likely to be due only to differences in patient populations and/or regional antimicrobial resistance, we believe our findings remain valid despite these limitations. Moreover, DDD and RDD analyses offer the possibility of longitudinal surveillance of overall antibiotic consumption and/or particular antibiotic classes, assuming there to be a constant case mix [29, 59, 69]. Additional patient-level analyses in the form of point or period prevalence surveys (PPS) in the participating hospitals including information on the patient level (e.g., patient age, weight, underlying diseases) and antibiotic treatment (indication, length, dosage, documentation) could be used to confirm the data continuously assessed by analysis of pharmacy dispensing data and help us to get a better picture of ACD in pediatric care. This also would allow us to draw more specific conclusions regarding appropriateness of prescribed antibiotic treatments.

To conclude, despite the limitations of the DDD/100 method in a pediatric setting, our study indicates inappropriate use of antibiotics at several levels: (1) high variability in ACD between hospitals of similar size and service type; (2) the extensive use of antibiotics belonging to the WHO “Watch” and “Reserve” categories. Both topics should be addressed by antimicrobial stewardship activities. Longitudinal surveillance of the relatively easily accessible pharmacy dispensing data allows to assess the development of ACD and the effectiveness of AMS interventions on a national level. In addition, patient level analyses are needed to confirm and to complement the data regarding prescribing quality to specify existing AMS activities. The pipeline for new antibiotics has run dry. It is urgent that currently available antibiotics are used prudently [9, 12, 13].

### Supplementary Information

Below is the link to the electronic supplementary material.

Supplementary file1 (DOCX 56 KB)

## Data Availability

The data that support the findings of this study are available from the ADKA-if-DGI project upon reasonable request.
