# Supplementary material for: Antibiotic use in pediatric acute care hospitals: an analysis of antibiotic consumption data from Germany, 2013–2020
Source: Infection. 2023 Nov 2;52(3):825–37. doi: 10.1007/s15010-023-02112-w (PMC11143023; doi:10.1007/s15010-023-02112-w)
Supplement: Supplementary file 1 — Supplementary file1 (DOCX 56 KB) [file 15010_2023_2112_MOESM1_ESM.docx]

**Supplementary Table 1:** Systematic of antibiotic substances

**Supplementary Table 2:** Overall antibiotic use density and of specific antibiotic classes (in DDD/100 pd and RDD/100 pd) in German pediatric acute care hospitals 2020.

|  | **DDD/100 pd** | | | **RDD/100 pd** | | |
| --- | --- | --- | --- | --- | --- | --- |
| **Antibiotic class** | Median | Interquartile range | | Median | Interquartile range | |
| **All systemic antibiotics** | 26.71 | 20.06- | 36.28 | 20.73 | 15.02- | 26.39 |
| **1st/2nd generation cephalosporins** | 5.88 | 3.90- | 9.29 | 3.49 | 2.47- | 5.16 |
| **Narrow-spectrum penicillins** | 3.59 | 2.51- | 5.08 | 1.65 | 1.36- | 2.62 |
| **3rd/4th generation cephalosporins** | 3.53 | 2.09- | 4.92 | 2.76 | 1.72- | 3.83 |
| **Aminopenicillin + BLI** | 2.63 | 1.50- | 4.02 | 2.48 | 1.50- | 3.92 |
| **Macrolides / clindamycin** | 2.05 | 1.40- | 3.00 | 1.32 | 0.93- | 2.01 |
| **Aminoglycosides** | 0.96 | 0.47- | 1.88 | 0.96 | 0.47- | 1.88 |
| **Metronidazole** | 0.76 | 0.40- | 1.50 | 0.76 | 0.40- | 1.50 |
| **Broad-spectrum penicillins** | 0.68 | 0.22- | 1.85 | 0.80 | 0.26- | 2.16 |
| **Glycopeptides** | 0.51 | 0.11- | 1.59 | 0.48 | 0.11- | 1.59 |
| **Carbapenems** | 0.46 | 0.21- | 1.57 | 0.46 | 0.21- | 1.57 |
| **Folate antagonists** | 0.42 | 0.15- | 1.03 | 0.42 | 0.15- | 1.03 |
| **Tetracyclines** | 0.28 | 0.05- | 0.63 | 0.15 | 0.03- | 0.32 |
| **Fluoroquinolones** | 0.24 | 0.08- | 0.51 | 0.24 | 0.08- | 0.51 |
| **Linezolid** | 0.00 | 0.00- | 0.06 | 0.00 | 0.00- | 0.06 |
| **Penicillins overall** | 7.61 | 5.82- | 10.55 | 6.00 | 4.48- | 7.68 |
| **Cephalosporins overall** | 9.78 | 7.28- | 13.61 | 6.57 | 5.03- | 9.35 |
| pd, patient-days |  |  |  |  |  |  |

**Supplementary Table 3:** Comparison of antibiotic use density in RDD/100 pd between different wards (regular, ICU and hematology-oncology) in hospitals of different size (<400, 400-800, >800 beds) and of different categories (non-university and university hospitals) in 2020. *Wilcoxon rank sum test.

|  |  | **Non-University hospitals** | | |  | **University hospitals** | | | **p-value*** |
| --- | --- | --- | --- | --- | --- | --- | --- | --- | --- |
|  | n | Median | Interquartile  range | | n | Median | Interquartile range | |  |
|  |  |  |  |  |  |  |  |  |  |
| **Regular wards** | **90** | **21.00** | **16.69-** | **24.57** | **21** | **23.64** | **18.08-** | **31.31** | 0.0758 |
| <400 beds | 36 | 19.91 | 13.56- | 22.84 |  |  |  |  |  |
| 400-800 beds | 35 | 20.56 | 16.55- | 25.59 |  |  |  |  |  |
| >800 beds | 19 | 22.18 | 19.25- | 25.18 | 21 | 23.64 | 18.08- | 31.31 | 0.7683 |
|  |  |  |  |  |  |  |  |  |  |
| **ICU** | **53** | **11.19** | **5.49-** | **20.23** | **22** | **32.28** | **26.65-** | **43.69** | **<0.0001** |
| <400 beds | 11 | 5.49 | 4.11- | 8.81 |  |  |  |  |  |
| 400-800 beds | 27 | 12.17 | 7.86- | 19.80 |  |  |  |  |  |
| >800 beds | 15 | 16.13 | 8.66- | 24.65 | 22 | 32.28 | 26.65- | 43.69 | **0.0002** |
|  |  |  |  |  |  |  |  |  |  |
| **Hematology-Oncology** | **3** | 29.87 | 22.87- | 37.95 | **18** | 67.28 | 59.32- | 86.40 | **0.0123** |
| pd, patient-days |  |  |  |  |  |  |  |  |  |

**Supplementary Table 4:** The five most commonly used antibiotic classes on different wards in non-university and university hospitals as DDD/100 pd (Median and Interquartile range [IQR]) in 2020.

|  | **All wards** | | | | **Regular ward** | | | | **ICU** | | | | **Hematology-oncology** | | | |
| --- | --- | --- | --- | --- | --- | --- | --- | --- | --- | --- | --- | --- | --- | --- | --- | --- |
| **All hospitals** |  | Median | IQR | |  | Median | IQR | |  | Median | IQR | |  | Median | IQR | |
|  | 1st/2nd gen. Cephalosporins | 5.88 | 3.90- | 9.29 | 1st/2nd gen. Cephalosporins | 7.07 | 4.53- | 10.49 | Narrow-spectrum pen. | 3.38 | 1.69- | 5.40 | Folate antagonists | 12.74 | 8.26- | 14.95 |
|  | Narrow-spectrum pen. | 3.59 | 2.51- | 5.08 | 3rd/4th gen. Cephalosporins | 3.91 | 2.36- | 5.29 | Glycopeptides | 2.28 | 0.59- | 5.91 | Broad-spectrum pen. | 8.84 | 2.07- | 14.76 |
|  | 3rd/4th gen. Cephalosporins | 3.53 | 2.09- | 4.92 | Narrow-spectrum pen. | 3.51 | 2.55- | 5.10 | 3rd/4th gen. Cephalosporins | 2.02 | 1.01- | 3.89 | Carbapenems | 8.65 | 6.33- | 10.43 |
|  | Aminopenicillins/BLI* | 2.63 | 1.50- | 4.02 | Aminopenicillins/BLI* | 3.01 | 1.77- | 4.88 | Carbapenems | 1.81 | 0.34- | 4.37 | Glycopeptides | 8.27 | 5.02- | 12.70 |
|  | Macrolides/clindamycin | 2.05 | 1.40- | 3.00 | Macrolides/clindamycin | 2.05 | 1.39- | 3.18 | Aminoglycosides | 1.71 | 0.82- | 2.54 | 3rd/4th gen. Cephalosporins | 5.23 | 2.64- | 11.74 |
|  | Cephalosporins | 9.78 | 7.28- | 13.61 | Cephalosporins | 11.27 | 8.30- | 15.74 | Penicillins | 6.76 | 3.83- | 10.78 | Penicillins overall | 12.88 | 6.97- | 16.40 |
|  | Penicillins | 7.61 | 5.82- | 10.55 | Penicillins | 8.18 | 5.82- | 10.60 | Cephalosporins | 3.98 | 1.97- | 7.81 | Cephalosporins overall | 8.10 | 6.02- | 18.11 |
|  |  |  |  |  |  |  |  |  |  |  |  |  |  |  |  |  |
| **Non-university hospitals** |  | Median | IQR | |  | Median | IQR | |  | Median | IQR | |  | Median | IQR | |
|  | 1st/2nd gen. Cephalosporins | 6.06 | 3.96- | 9.72 | 1st/2nd gen. Cephalosporins | 7.34 | 4.77- | 11.25 | Narrow-spectrum pen. | 3.24 | 1.55- | 4.80 | Folate antagonists | 10.33 | 5.33- | 12.47 |
|  | Narrow-spectrum pen. | 3.59 | 2.54- | 5.10 | 3rd/4th gen. Cephalosporins | 3.92 | 2.14- | 5.22 | 3rd/4th gen. Cephalosporins | 1.51 | 0.67- | 2.87 | 3rd/4th gen. Cephalosporins | 9.02 | 5.76- | 10.38 |
|  | 3rd/4th gen. Cephalosporins | 3.50 | 1.91- | 4.68 | Narrow-spectrum pen. | 3.53 | 2.62- | 5.25 | Aminoglycosides | 1.50 | 0.91- | 2.53 | Aminoglycosides | 3.09 | 2.69- | 4.26 |
|  | Aminopenicillins/BLI* | 2.67 | 1.67- | 4.11 | Aminopenicillins/BLI* | 2.99 | 2.09- | 4.83 | 1st/2nd gen. Cephalosporins | 0.92 | 0.41- | 1.85 | Macrolides/clindamycin | 2.82 | 2.16- | 2.84 |
|  | Macrolides/clindamycin | 2.05 | 1.38- | 3.12 | Macrolides/clindamycin | 2.08 | 1.48- | 3.44 | Glycopeptides | 0.89 | 0.26- | 2.50 | 1st/2nd gen. Cephalosporins | 2.11 | 1.88- | 4.23 |
|  | Cephalosporins | 9.69 | 7.35- | 13.76 | Cephalosporins | 11.41 | 8.32- | 16.26 | Penicillins | 4.98 | 2.93- | 8.23 | Cephalosporins | 11.13 | 7.64- | 14.62 |
|  | Penicillins | 7.30 | 5.36- | 9.89 | Penicillins | 7.87 | 5.82- | 10.02 | Cephalosporins | 3.09 | 1.43- | 4.36 | Penicillins | 3.44 | 3.18- | 5.25 |
|  |  |  |  |  |  |  |  |  |  |  |  |  |  |  |  |  |
| **University hospitals** |  | Median | IQR | |  | Median | IQR | |  | Median | IQR | |  | Median | IQR | |
|  | 1st/2nd gen. Cephalosporins | 5.18 | 3.60- | 7.23 | 1st/2nd gen. Cephalosporins | 5.83 | 3.92- | 7.72 | Glycopeptides | 6.25 | 3.08- | 8.29 | Folate antagonists | 12.87 | 8.31- | 17.42 |
|  | 3rd/4th gen. Cephalosporins | 4.14 | 2.89- | 6.66 | 3rd/4th gen. Cephalosporins | 3.89 | 2.96- | 6.49 | Carbapenems | 5.49 | 3.51- | 6.72 | Broad-spectrum penicillins | 10.16 | 6.19- | 14.84 |
|  | Broad-spectrum penicillins | 3.53 | 2.28- | 4.60 | Narrow-spectrum pen. | 3.44 | 2.49- | 4.15 | 1st/2nd gen. Cephalosporins | 4.53 | 2.44- | 6.82 | Glycopeptides | 9.78 | 6.32- | 14.20 |
|  | Carbapenems | 3.45 | 1.96- | 4.93 | Aminopenicillins/BLI* | 3.14 | 1.16- | 5.23 | Narrow-spectrum pen. | 4.10 | 1.97- | 7.17 | Carbapenems | 9.06 | 6.77- | 10.69 |
|  | Narrow-spectrum pen. | 3.36 | 2.49- | 4.89 | Broad-spectrum penicillins | 2.22 | 0.91- | 3.08 | 3rd/4th gen. Cephalosporins | 3.93 | 2.40- | 6.02 | 3rd/4th gen. Cephalosporins | 5.06 | 2.97- | 15.02 |
|  | Cephalosporins | 10.86 | 6.73- | 13.20 | Cephalosporins | 9.99 | 7.67- | 14.47 | Penicillins | 10.07 | 8.55- | 14.65 | Penicillins | 13.67 | 8.27- | 16.50 |
|  | Penicillins | 9.65 | 7.75- | 12.13 | Penicillins | 9.01 | 6.20- | 11.98 | Cephalosporins | 8.38 | 6.36- | 12.35 | Cephalosporins | 7.96 | 6.26- | 20.63 |
|  | pd, patient-days; DDD, defined daily doses; IQR, interquartile range; ICU, intensive care unit; gen., generation; pen., penicillins | | | | | | | | | | | | | | | |
